# Bacteriocin-Like Inhibitory Substances in Staphylococci of Different Origins and Species With Activity Against Relevant Pathogens

**DOI:** 10.3389/fmicb.2022.870510

**Published:** 2022-04-26

**Authors:** Rosa Fernández-Fernández, Carmen Lozano, Paula Eguizábal, Laura Ruiz-Ripa, Sandra Martínez-Álvarez, Idris Nasir Abdullahi, Myriam Zarazaga, Carmen Torres

**Affiliations:** Area of Biochemistry and Molecular Biology, OneHealth-UR Research Group, University of La Rioja, Logroño, Spain

**Keywords:** bacteriocin, multidrug resistant (MDR) bacteria, BLIS, coagulase-negative staphylococci, coagulase-positive staphylococci

## Abstract

Bacteriocins are antimicrobial peptides with relevance in the modulation of human and animal microbiota that have gained interest in biomedical and biotechnological applications. In this study, the production of bacteriocin-like inhibitory substances (BLIS) was tested among a collection of 890 staphylococci of different origins (humans, animals, food, and the environment) and species, both coagulase-positive (CoPS, 238 isolates of 3 species) and coagulase-negative staphylococci (CoNS, 652 isolates of 26 species). Of the 890 staphylococci, 60 (6.7%) showed antimicrobial activity by the *spot-on-lawn* method against at least one of the 25 indicator bacteria tested. BLIS-producer (BLIS^+^) isolates were detected in 8.8% of CoPS and 6.0% of CoNS. The staphylococcal species with the highest percentages of BLIS^+^ isolates were *S. chromogenes* (38.5%), *S. pseudintermedius* (26.7%), and *S. warneri* (23.1%). The production of BLIS was more frequently detected among isolates of pets, wild animals, and food. Moreover, 13 BLIS^+^ isolates showed wide antimicrobial activiy spectrum, and 7 of these isolates (of species *S. aureus*, *S. pseudintermedius*, *S. sciuri*, and *S. hominis*) demonstrated antimicrobial activity against more than 70% of the indicator bacteria tested. The genetic characterization (by PCR and sequencing) of the 60 BLIS^+^ isolates revealed the detection of (a) 11 CoNS and CoPS isolates carrying putative lantibiotic-like genes; (b) 3 *S. pseudintermedius* isolates harboring the genes of BacSp222 bacteriocin; and (c) 2 *S. chromogenes* isolates that presented the gene of a putative cyclic bacteriocin (uberolysin-like), being the first report in this CoNS species. Antimicrobial susceptibility testing was performed in BLIS^+^ isolates and one-third of the CoNS isolates showed susceptibility to all antibiotics tested, which also lacked the virulence genes studied. These BLIS^+^ CoNS are good candidates for further characterization studies.

## Introduction

*Staphylococcus* is a genus widely distributed in the environment and comprises important members of the major bacterial communities colonizing the skin and mucous membranes of humans and animals (Kranjec et al., 2020). This genus comprises a high diversity of species, traditionally separated into two major groups based on their ability to clot the plasma: coagulase-positive staphylococci (CoPS) and coagulase-negative staphylococci (CoNS). CoNS is the broadest group, with more than 80% of species (Becker et al., 2015).

Although staphylococci often maintain a commensal or symbiotic relationship with their hosts, staphylococcal infections could develop in specific cases (de Freire Bastos et al., 2020). Some staphylococcal species are more related to human and animal infectious diseases, being *Staphylococcus aureus* one of the most important.

Individual bacterial strains living in highly competitive and polymicrobial environments have developed multiple types of interaction and self-defense mechanisms. The survival success can include different capabilities such as a higher specificity to a limited number of host attachment sites, a better ability to take up and metabolize micronutrients, or the production of antibacterial substances that inhibit competitors (Janek et al., 2016). The most widely distributed microbial defense mechanisms are bacteriocins, in most cases ribosomally synthesized peptides with antibacterial properties (França et al., 2021). From an ecological point of view, bacteriocins are synthesized to confer a selective advantage to the producer in terms of niche colonization ability since these molecules often display activity against closely related bacterial species (Heilbronner et al., 2021).

Staphylococci are no exception with respect to antimicrobial substances production. There is a wide variety of staphylococcal bacteriocins, highlighting the lantibiotic group (Class I), characterized by their post-translationally modifications, usually by enzymatic tailoring (Heilbronner et al., 2021). Moreover, other well-described antimicrobial peptides include those that remain unaltered (Class II bacteriocins), the heat-labile proteins differentiated between lytic and non-lytic bacteriocins (Class III) (Heng et al., 2007), and the cyclic molecules (Class IV) (de Freire Bastos et al., 2020; Newstead et al., 2020). Other antimicrobial peptides have recently been discovered in staphylococci isolates, such as those included in the sactipeptide group (de Freire Bastos et al., 2020) and the well-known fibupeptide lugdunin (a major non-ribosomal peptide, NRP) described by Zipperer et al. (2016). Moreover, bacteriocins previously described in other genera, such as the thiopeptide Micrococcin P1, have been also detected in staphylococci (Carnio et al., 2000). In addition, there is another type of antimicrobial substance known as bacteriocin-like inhibitory substances (BLIS), which are not obtained in pure form or fully characterized, and they have also been reported in staphylococci (James and Tagg, 1991; Mak, 2018).

*Staphylococcus aureus* is considered the most relevant CoPS in terms of bacteriocin production (generally, class II type bacteriocins), although CoNS bacteriocins (mostly lantibiotics) have been also reported in the literature (Bastos et al., 2009). Bacteriocin produced by staphylococci are active against pathogenic staphylococci, including methicillin-resistant *S. aureus* (Kranjec et al., 2020). These antimicrobial compounds could be of interest as food additives, therapeutic agents, or modulators of the microbiota (Soltani et al., 2021).

Due to the growing interest in the identification and characterization of new antimicrobial agents, this study sought to determine the production of BLIS in a large collection of CoNS and CoPS isolates of different origins (human, animal, food, and the environment) and to characterize the BLIS-producer (BLIS^+^) isolates at the genomic level to determine the presence of bacteriocin encoding genes as well as antimicrobial resistance (AMR) and virulence genes.

## Materials and Methods

### Bacterial Collection

A total of 890 *Staphylococcus* spp. isolates, both CoPS (*n* = 238, of three different species) and CoNS (*n* = 652, of 26 different species), recovered from diverse origins (humans, *n* = 27; food, *n* = 278; wild animals, *n* = 403; pets, *n* = 50; and the environment, *n* = 132), were included in this study. All isolates of humans and animals were obtained from nasal samples of healthy individuals (except 4 CoPS clinical isolates of humans) and were identified using matrix-assisted laser desorption/ionization time of flight mass spectrometry (MALDI-TOF, Bruker). Table 1 shows the species and origins of the isolates tested, and those showing antimicrobial activity. The isolates were obtained from previous studies performed by the OneHealth-UR research group of the University of La Rioja, and many of them were included in previously published papers (Gómez-Sanz et al., 2013; Gómez et al., 2017; Lozano et al., 2017; Mama et al., 2019; Ruiz-Ripa et al., 2019, 2020, 2021).

**TABLE 1:** Species, origins, and bacteriocin-like inhibitory substances (BLIS) production capacity of the 890 CoPS and CoNS isolates included in this study.

Type of *Staphylococci*	Species	Number of isolates of different origins tested/number of BLIS^+^ isolates (%)					
		Total isolates	Human	Food	Wild animal	Pet	Environmental
CoPS	*S. aureus*	147/5 (3.4%)	11/0	72/2 (2.8%)	57/2 (3.5%)	0	7/1 (14.3%)
	*S. pseudintermedius*	60/16 (26.7%)	9/1 (11.1%)	1/0	0	50/15 (30%)	0
	*S. delphini*	31/0	0	19/0	12/0	0	0
	Total CoPS*	238/21 (8.8)	20/1 (5%)	92/2 (2.2%)	69/2 (2.9%)	50/15 (30%)	7/1 (14.3%)
CoNS	*S. sciuri*	214/13 (3.1%)	0	23/2 (8.7%)	182/11 (6%)	0	9/0
	*S. saprophyticus*	69/0	0	39/0	5/0	0	25/0
	*S. lentus*	48/0	0	16/0	28/0	0	4/0
	*S. xylosus*	48/3 (6.3%)	0	7/0	24/3 (12.5%)	0	17/0
	*S. epidermidis*	38/4 (10.5%)	4/0	17/4 (23.5%)	5/0	0	12/0
	*S. fleurettii*	29/0	0	14/0	15/0	0	0
	*S. chromogenes*	26/10 (38.5%)	0	7/3 (42.8%)	17/7 (41.17%)	0	2/0
	*S. warneri*	26/6 (23.1%)	1/0	24/6 (25%)	0	0	1/0
	*S. vitulinis*	24/0	0	5	19	0	0
	*S. simulans*	22/1 (4.5%)	0	16/0	3/1 (33.3%)	0	3/0
	*S. arlettae*	21/0	0	0	0	0	21/0
	*S. cohnii*	16/0	0	2/0	2/0	0	12/0
	*S. equorum*	16/0	0	2/0	13/0	0	1/0
	*S. pasteuri*	9/0	0	8/0	0	0	1/0
	*S. hominis*	8/1 (12.5%)	1/0	3/0	1/0	0	3/1 (33.3%)
	*S. capitis*	6/0	0	1/0	1/0	0	4/0
	*S. hyicus*	6/1 (16.7%)	0	2/0	4/1 (25%)	0	0
	*S. succinus*	6/0	0	0	5/0	0	1/0
	*S. haemolyticus*	5/0	0	0	2/0	0	3/0
	*S. nepalensis*	5/0	0	0	0	0	5/0
	*S. kloosii*	3/0	0	0	3/0	0	0
	*S. schleiferi*	3/0	0	0	3/0	0	0
	*S. auricularis*	1/0	0	0	0	0	1/0
	*S. felis*	1/0	0	0	1/0	0	0
	*S. lugdunensis*	1/0	1/0	0	0	0	0
	*S. simiae*	1/0	0	0	1/0	0	0
	Total CoNS*	652/39 (6%)	7/0	186/15 (8.1%)	334/23 (6.9%)	0	125/1 (0.8%)
Total (CoPS + CoNS)*		890/60 (6.7%)	27/1 (3.7%)	278/17 (6.1%)	403/25 (6.2%)	50/15 (30%)	132/2 (1.5%)

*Statistically significant differences with p ≤ 0.05, depending on the origin of the isolates.

### Screening of Antimicrobial Activity

The screening for the production of BLIS was performed with the complete staphylococci collection (*n* = 890 isolates) in agar diffusion assays by the *spot-on-lawn* method (Supplementary Figure 1). For that purpose, 25 indicator bacteria of different genera and species were used. Gram-positive and Gram-negative bacteria of 8 different genera (*Staphylococcus*, *Enterococcus*, *Micrococcus*, *Streptococcus*, *Clostridium*, *Listeria*, *Escherichia*, and *Pseudomonas*) and 19 different species, including multidrug-resistant bacteria and relevant pathogens, were used as indicator bacteria (Table 2).

**TABLE 2:** Indicator bacteria used in the antimicrobial test, differentiated in terms of genera and species.

Type of bacteria	Genera	Indicator Bacteria (*n* = 25)^a^	UR-Reference
Gram+	*Staphylococcus*	MRSA (2)	C1570, C5313
		MSSA	C411
		MRSP	C2381
		MSSP	C3468
		*S. delphini*	C9459
		*S. epidermidis*	C2663
		*S. haemolyticus*	C2709
		*S. lugdunensis*	C10107
		*S. sciuri*	C9780
	*Enterococcus*	*E. casseliflavus*	C1232
		*E. durans vanA*	C1433
		*E. faecalis*	C410
		*E. faecium vanA*	C2321
		*E. gallinarum*	C2310
		*E. hirae vanA*	C1436
	*Micrococcus*	*M. luteus*	C157
	*Listeria*	*L. monocytogenes*	C137
	*Streptococcus*	*S. suis* (4)	X2057, X2058, X2060, X2061
	*Clostridium*	*C. perfringens*	X2967
Gram−	*Escherichia*	*E. coli*	C408
	*Pseudomonas*	*P. aeruginosa*	C3282

*^a^MRSA, methicillin-resistant S. aureus; MSSA, methicillin-susceptible S. aureus; MRSP, methicillin-resistant S. pseudintermedius; MSSP, methicillin-susceptible S. pseudintermedius; vanA, acquired mechanism of vancomycin resistance.*

#### Agar Diffusion Assays (*Spot-On-Lawn* method)

All staphylococci to be tested for the production of BLIS and the 25 indicator isolates were grown on brain heart infusion (BHI) agar (Condalab, Spain) for 24 h at 37°C. Indicator isolates were resuspended in BHI broth at a concentration equivalent to 0.5 MacFarland turbidity. Then, 10 μl of each indicator bacteria were added to 5 ml of semisolid tryptic soy broth (SS-TSB) (BD, Difco, France) media supplemented with 0.3% of yeast extract and 0.7% of bacteriological agar (autoclaved and cooled until 45°C). After mixing, the SS-TSB media with the indicator bacteria were poured into a tryptic soy agar (TSA) plate (BD, Difco, France), which was also supplemented with 0.3% of yeast extract. Staphylococci to be tested for the production of BLIS were spotted on prepared plates with each of the indicator isolates and were incubated in aerobic conditions for 24 h at 37°C. Anaerobic conditions were used when the indicator isolate was *Clostridium perfringens*, and Columbia agar with 5% sheep blood (bioMérieux, France) was used instead of TSA when indicator isolate was *Streptococcus suis*. Inhibition halos were checked and measured in millimeters (mm).

#### DNA Isolation

A colony of fresh culture bacteria was resuspended in 45 μl of sterile Milli-Q water and 5 μl of lysostaphin (1 mg/ml). After incubation (10 min, 37°C), 45 μl of Milli-Q water, 150 μl of Tris-HCl (0.1 M, pH = 8), and 5 μl of proteinase K (2 mg/ml) were added. Finally, the total volume was incubated (10 min, 60°C), boiled (5 min, 100°C), and centrifuged (3 min, 12,000 rpm). The supernatant was used for PCR assays.

#### Detection of Bacteriocin Encoding Genes in BLIS^+^ Isolates

The presence of 23 bacteriocin structural genes was tested by PCR and sequencing in the 60 BLIS^+^ isolates (*aurA*, *aucA*, *epiA*, *sacaA/sacbA*, *gdmA*, *bacSp222*, *nsj*, *hyiA*, *hycS*, *bacCH91*, *bsaA2*, *lugD*, *acIA*, *ale-1*, *lss*, *nukA*, *nkqA*, *eciA*, *pepA*, *elxA*, *elkA*, *ecdA*, *orf4*) and also the precursor gene for the NRP lugdunin (*lugD*). Moreover, three bacteriocin gene families were considered based on the nucleotide sequences of some bacteriocin structural genes with high similarities. These families were BS (BsaA2 and BacCH91), GEST (Gallidermin, Epidermin, and Staphylococcin T), and NUK (Nukacin KQU-131, Nukacin 3299, and Nukacin ISK1). Primers were designed for the detection of some bacteriocins and the bacteriocin families. The primer design was made with the online Primer3 software (Whitehead Institute for Biomedical Research, Cambridge, MA, United States) (Untergasser et al., 2012). To optimize primer creation, bacteriocin gene and peptide sequences were analyzed using Geneious and MegaX (refer to Supplementary Table 1 for primer sequences and PCR conditions).

### Characterization of Antimicrobial Resistance, Virulence, and Molecular Typing of BLIS^+^ Isolates

The antimicrobial susceptibility testing, the detection of antibiotic resistance and virulence genes, and/or the molecular typing (*spa*, MLST, and/or *agr*) of 43 of the 60 BLIS^+^ isolates included in this study were performed in previous studies (Supplementary Table 2). The initial characterization of these isolates was used and partially completed in this study. The remaining 17 BLIS^+^ isolates were completely characterized in this study based on the criteria indicated in the following sections.

#### Antimicrobial Resistance Phenotype/Genotype

The susceptibility to 13 antimicrobial agents was evaluated in the BLIS^+^ isolates, including the following antibiotics: penicillin, cefoxitin/oxacillin, erythromycin, clindamycin, gentamicin, tobramycin, streptomycin, tetracycline, ciprofloxacin, chloramphenicol, linezolid, trimethoprim–sulfamethoxazole, and fusidic acid. The disk diffusion results for all antimicrobial agents were interpreted using the European Committee on Antimicrobial Susceptibility Testing criteria (EUCAST, 2021).

The presence of the following resistance genes was tested by single PCR according to the corresponding phenotypes of AMR: *bla*Z, *mec*A, *mec*B, *mec*C, *erm*(A), *erm*(B), *erm*(C), *erm*(T), *msr*(A), *mph*(C), *Inu*(A), *Inu*(B), *vga*(A), *sal*(A), *aac(6′)-Ie-aph(2″)-Ia*, *ant*(4′)-Ia, *str*, *ant*(6), *tet*(L), *tet*(M), *tet*(K), *fex*(A), *fex*(B), *cat*_pC194_, *cat*_pC221_, *cat*_pC223_, *fus*B, *fus*C, *fus*D, *dfr*A, *dfr*D, *dfr*G, and *dfr*K (Ruiz-Ripa et al., 2020).

Multidrug resistance (MDR) was considered when staphylococci presented resistance to at least three different families of antibiotics. In this sense, clindamycin resistance due to the intrinsic presence of the *sal*(A) gene in *S. sciuri* was not considered for MDR classification, unless an additionally acquired resistance gene was found (Ruiz-Ripa et al., 2020).

#### Virulence Factors

The presence of relevant virulence genes, such as the leukocidin genes *luk*SF-PV, *luk*M, *luk*ED, and *luk*PQ, the toxic shock syndrome toxin 1 (*tst*), and the exfoliative toxins A, B, and D (*eta*, *etb*, and *etd*), respectively, was studied by single PCR and confirmed by amplicon sequencing in all CoNS and *S. aureus* isolates. *S. pseudintermedius* isolates were screened for the presence of the leukocidin gene *luk*S/F-I, the exfoliative genes *siet*, *expA*, *and expB*, and the enterotoxin genes *si-ent* and *sec*-canine by PCR (Gómez-Sanz et al., 2013).

Positive controls from the collection of the University of La Rioja were included in all PCR assays.

#### Molecular Typing

*Spa*-typing was carried out by PCR and amplicon sequencing in all *S. aureus* isolates. The *spa* sequences were analyzed using Ridom Staph-Type software version 1.5.21 (Ridom GmbH, Münster, Germany). Multilocus sequence typing (MLST) was performed on *S. aureus* and *S. pseudintermedius* isolates, as well as on a selected *S. epidermidis* isolate (Bannoehr et al., 2007; Ruiz-Ripa et al., 2019). All isolates carrying the *mecA* gene were subjected to SCC*mec* typing by multiplex PCRs, and *agr*-typing was characterized following standard methodology in all *S. aureus* and *S. pseudintermedius* isolates (Zhang et al., 2005; Perreten et al., 2010).

#### Statistical Analysis

The Pearson’s chi-square test was used to explore significant differences in the antimicrobial activity of the isolates tested. Comparisons between BLIS production and origins (human, food, wild animal, pet, and environment) were carried out in the total collection of 890 staphylococci, as well as in the CoPS and CoNS isolates, considered in a separate way. Moreover, the relationship between the rate of BLIS production and the staphylococcal species was studied. Analyses were carried out using SPSS statistical software version 26.0 (IBM^®^, SPSS Inc., Chicago, IL, United States) and significance was set at *p* ≤ 0.05.

## Results

### Bacteriocin-Like Production in Isolates of Different Species and Origins

**FIGURE 1:**
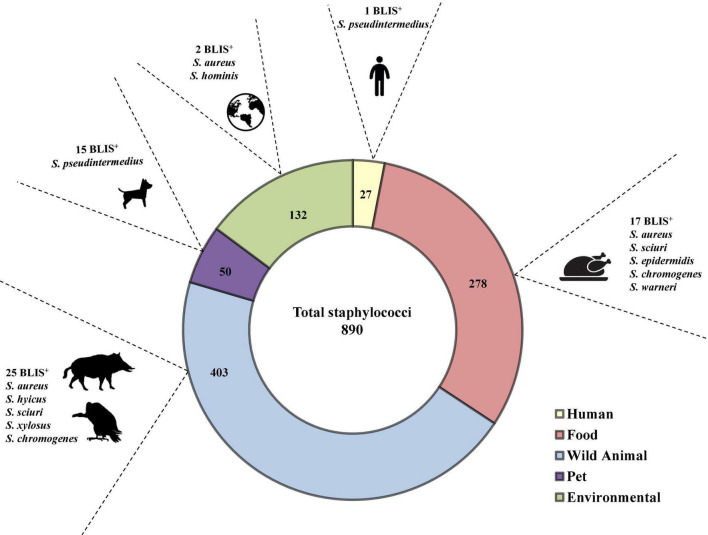
Distribution of the 60 BLIS^+^ isolates per origin, indicating the species and the number of isolates in which antimicrobial activity was detected.

Bacteriocin-like production assays performed using the spot on the lawn method revealed that 60 out of 890 isolates tested (6.7%) showed antimicrobial activity (BLIS^+^) against at least one of the 25 indicator isolates (Table 1). Differences were observed in the percentage of BLIS^+^ isolates between CoPS (*n* = 21 of 238 isolates, 8.8%) and CoNS (*n* = 39 of 652 isolates, 6%), and among staphylococcal species (number of BLIS^+^ isolates/total number of tested isolates): *S. aureus* (5/147), *S. pseudintermedius* (16/60), *S. sciuri* (13/214), *S. hyicus* (1/6), *S. hominis* (1/8), *S. chromogenes* (10/26), *S. epidermidis* (4/38), *S. warneri* (6/26), *S. xylosus* (3/48), and *S. simulans* (1/22). No BLIS^+^ isolates were detected among the remaining staphylococcal species. The comparison of the rates of BLIS^+^ isolates by staphylococcal species showed statistically significant differences, and the higher rates were detected for the following species: *S. chromogenes* (38.5%), *S. pseudintermedius* (26.7%), and *S. warneri* (23.1%). When the origin of the isolates and the rate of BLIS^+^ isolates were considered, statistically significant differences were observed for the total collection of staphylococci, as well as for the collections of CoPS and CoNS (*p* ≤ 0.05). The highest frequency of BLIS^+^ isolates was found in isolates obtained from pets (*n* = 15, 30%), followed by those of wild animals (*n* = 25, 6.2%) and of food samples (*n* = 17, 6.11%) (Table 1 and Figure 1).

### Antimicrobial Activity of BLIS^+^ Isolates

The antimicrobial profile of the 60 BLIS^+^ isolates against the 25 indicator bacteria is summarized in Table 3. Antimicrobial activity was detected against all the species of Gram-positive indicator bacteria tested; nevertheless, none of the BLIS^+^ isolates showed activity against the Gram-negative indicator bacteria used in this study (*Escherichia coli* and *Pseudomonas aeruginosa*). The indicator bacteria with the highest inhibition rates were as follows: *S. delphini* (inhibited by 62% of BLIS^+^ isolates), *S. pseudintermedius* (55%), *M. luteus* (38%), and *C. perfringens* (35%).

**TABLE 3:** Antimicrobial activity of the 60 bacteriocin-like inhibitory substances producer (BLIS^+^) isolates against 25 indicator bacteria.

Indicator bacteria (No isolates)^a^	No of BLIS^+^ isolates^b^										
	*S. pseudintermedius* (16)	*S. aureus* (5)	*S. sciuri* (13)	*S. chromogenes* (10)	*S. warneri* (6)	*S. epidermidis* (4)	*S. xylosus* (3)	*S. hominis* (1)	*S. hyicus* (1)	*S. simulans* (1)	No/%^c^
MRSA (2)	10	2	2	2	0	0	0	1	0	1	18/30
MSSA	0	2	2	2	0	0	0	1	0	1	8/13
MRSP	7	1	2	0	1	1	0	1	0	0	13/22
MSSP	9	2	12	6	1	1	0	1	0	1	33/55
*S. epidermidis*	6	1	2	0	0	1	0	1	1	1	13/22
*S. delphini*	10	4	13	6	0	1	0	1	1	1	37/62
*S. haemolyticus*	0	0	0	0	1	1	0	0	0	1	3/5
*S. lugdunensis*	5	1	11	0	0	0	0	1	1	1	20/33
*S. sciuri*	3	2	2	6	0	1	0	1	0	1	16/27
*E. casseliflavus*	3	1	2	1	0	0	0	1	1	0	9/15
*E. durans vanA*	3	2	2	1	0	0	0	1	0	0	9/15
*E. faecalis*	7	1	2	0	0	0	0	1	0	0	11/18
*E. faecium vanA*	3	1	2	2	0	0	0	1	1	0	10/17
*E. gallinarum*	8	1	2	2	0	0	0	1	1	0	15/25
*E. hirae vanA*	6	2	2	2	0	0	0	1	1	0	14/23
*L. monocytogenes*	4	1	2	2	0	0	0	1	0	0	10/17
*M. luteus*	4	2	2	7	1	2	3	1	1	0	23/38
*S. suis* (4)	12	4	2	1	0	0	0	0	0	0	19/32
*C. perfringens*	5	3	2	1	6	2	0	1	1	0	21/35
*E. coli/P. aeruginosa*	0	0	0	0	0	0	0	0	0	0	0

*^a^MRSA, methicillin resistant S. aureus; MSSA, methicillin-susceptible S. aureus; MRSP, methicillin-resistant S. pseudintermedius; MSSP, methicillin-susceptible S. pseudintermedius; vanA, acquired mechanism of vancomycin resistance.*

*^b^The number of staphylococcal isolates that showed antimicrobial activity against each indicator bacteria is indicated.*

*^c^The number of the total staphylococcal isolates that showed antimicrobial activity against each indicator bacteria and percentage respect to the 60 BLIS^+^ isolates is represented.*

Moreover, our results reflected intense bioactivities both in CoPS and CoNS, detecting strains of some species (*S. pseudintermedius*, *S. aureus*, *S. hominis*, and *S. sciuri*) with antimicrobial activity against more than 70% of the 25 indicator bacteria tested. In addition, high diversity was found in the number of indicator isolates inhibited by the BLIS^+^ isolates of each staphylococcal species. For example, one BLIS^+^ *S. aureus* isolate was able to prevent the growth of up to 21 indicators, while two BLIS^+^ *S. aureus* isolates only inhibited the growth of two indicator isolates. In the remaining staphylococcal species, the following ranges of the number of indicator isolates inhibited were detected among BLIS^+^ isolates: *S. pseudintermedius* (1–18), *S. sciuri* (1–22), *S. chromogenes* (1–10), *S. epidermidis* (1–7), and *S. warneri* (1–5). In the case of the specie *S. xylosus*, the three BLIS^+^ isolates only showed antimicrobial activity against *M. luteus*.

**FIGURE 2:**
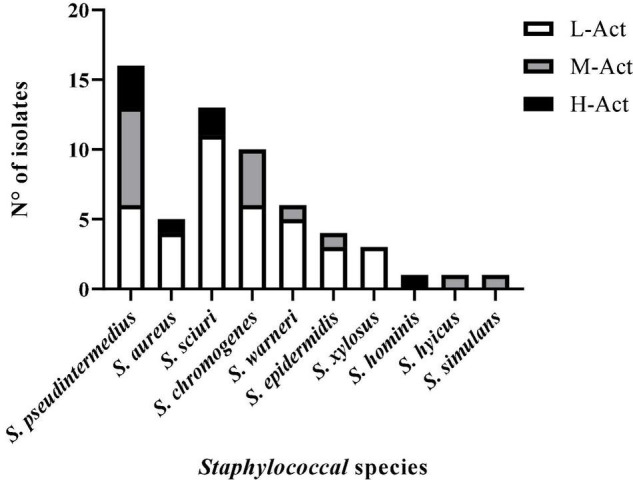
Antimicrobial activity level [low (L-Act), medium (M-Act), or high (H-Act)] of the 60 BLIS^+^ isolates (of 10 staphylococcal species) according to the percentage of indicator bacteria inhibited (L-Act: <20%; M-Act: 20–70%; H-Act: >70%).

For an overview of the antimicrobial activity of the 60 BLIS^+^ isolates, three levels of activity were established based on the percentage of indicator bacteria inhibited by each BLIS^+^ staphylococci: high activity (H-Act, activity against > 70% of the 25 indicator bacteria tested), medium activity (M-Act, 20–70%), and low activity (L-Act, <20%) (Figure 2). In all staphylococcal species with BLIS^+^ isolates, there were H-Act or M-Act BLIS^+^ isolates, except for *S. xylosus*. Moreover, BLIS^+^ isolates with H-Act were identified for the species *S. aureus* (20% of BLIS^+^ isolates), *S. pseudintermedius* (19% of BLIS^+^ isolates), *S. sciuri* (15% of BLIS^+^ isolates), and *S. hominis* (the unique BLIS^+^ isolate of this species).

A total of 13 isolates among the total collection of 60 BLIS^+^ isolates showed high or medium antimicrobial activities, and their antimicrobial profiles against relevant indicator bacteria are presented in Table 4. Some of these 13 BLIS^+^ isolates produced high inhibition halos (larger than 10 mm in diameter) against methicillin-resistant *S. pseudintermedius* (MRSP), methicillin-resistant *S. aureus* (MRSA), vancomycin-resistant enterococci (VRE), *L. monocytogenes*, *C. perfringens*, or *S. suis*, among others.

**TABLE 4:** Antimicrobial profile of the 13 BLIS^+^ isolates with high**/medium* antimicrobial activities^a^ against relevant indicator bacteria^b^.

Indicator bacteria (No isolates)^b^	No of indicator bacteria inhibited												
	*S. pseudintermedius*			*S. aureus*	*S. sciuri*		*S. hominis*	*S. chromogenes*		*S. warneri*	*S. epidermidis*	*S. hyicus*	*S. simulans*
	C8189**	C8478**	C8479**	C5802**	X3011**	X3041**	C5835**	C9838*	C9581*	X2969*	X3009*	C9585*	C9832*

	Human	Pet		Environmental	Food		Environmental	Wild animal		Food	Food	Wild animal	Wild animal
MR-*Staphylococcus* (3)	3	3	3	3	3	3	3	0	0	2	2	0	3
MS-*Staphylococcus* (7)	3	3	3	6	6	6	6	3	0	2	5	3	7
Total *Staphylococcus spp.* (10)	6	6	6	9	9	9	9	3	0	2	7	3	10
VR-*Enterococcus* (3)	3	3	3	3	3	3	3	3	2	0	0	2	0
VS-*Enterococcus* (3)	3	3	3	3	3	3	3	2	0	0	0	2	0
Total *Enterococcus* spp. (6)	6	6	6	6	6	6	6	5	2	0	0	4	0
*L. monocytogenes* (1)	1	1	1	1	1	1	1	1	0	0	0	0	0
*M. luteus* (1)	1	1	1	0	1	1	1	1	1	1	1	1	0
*S. suis* (4)	3	3	3	4	4	4	0	0	4	0	0	0	0
*C. perfringens* (1)	1	1	1	1	1	1	1	0	1	1	0	1	0
*E. coli* (1)*/P. aeruginosa* (1)	0	0	0	0	0	0	0	0	0	0	0	0	0
Total number/% of indicator bacteria inhibited	18/72%	18/72%	18/72%	21/84%	22/88%	22/88%	18/72%	10/40%	8/32%	5/20%	7/28%	9/36%	10/40%

^a^High activity** (>70%); medium activity* (20–70%) of the number of indicator bacteria inhibited.

*^b^MR, methicillin-resistant; MS, methicillin-susceptible; VR, vancomycin-resistant; VS, vancomycin-susceptible.*

### Bacteriocin Encoding Genes Detected in BLIS^+^ Isolates

The presence of 23 bacteriocin-encoding genes was analyzed by PCR and sequencing in the collection of 60 BLIS^+^ isolates. At least one bacteriocin gene was detected in 16 of these isolates. The characteristics of these isolates are shown in Table 5. The bacteriocin encoding genes detected were as follows: (1) lantibiotic-like (*n* = 11 isolates, five CoNS and six CoPS); (2) uberolysin-like (two CoNS isolates); and (3) the BacSp222 (three CoPS isolates). Ten of these staphylococci were susceptible to all antibiotics tested.

**TABLE 5:** Characteristics of the BLIS^+^ isolates that carry bacteriocin encoding genes.

Species	Strain	Origin	Antimicrobial level^a^	Bacteriocins detected	Antimicrobial resistance phenotype-[genotype]^b^	Virulence content	*spa-*MLST-CC/*agr*
*S. pseudintermedius*	C4502	Pet	M-Act	Lantibiotic-like	SXT-[*dfr*G]	*se-int, sea1, sed1 siet, luk*S/F-I	ST160/III
	C8189	Human	H-Act	Bacsp222	ERY-CLI-[*erm*(B)]	*luk*S/F-I,*siet*	ST241/III
	C8478	Pet	H-Act	Bacsp222	ERY-CLI-[*erm*(B)]	*luk*S/F-I,*siet*	ST241/III
	C8479	Pet	H-Act	Bacsp222	ERY-CLI-[*erm*(B)]	*luk*S/F-I,*siet*	ST241/III
*S. aureus*	C5802	Environment	H-Act	Lantibiotic-like	PEN-[*bla*Z]	*luk*MF′,*luk*ED,*etD2*	t843-ST130-CC130/III
	C6770	Wild animal	L-Act	Lantibiotic-like	Susceptible	−	t1125-CC5/II
	C8609	Wild animal	L-Act	Lantibiotic-like	Susceptible	−	t11225-CC425/II
	X3410	Food	L-Act	Lantibiotic-like	Susceptible	−	t10234/I
*S. chromogenes*	C9838	Wild animal	M-Act	Uberolysin-like	Susceptible	−	NS^c^
	C9581	Wild animal	M-Act	Uberolysin-like	Susceptible	−	NS^c^
	C9727	Wild animal	M-Act	Lantibiotic-like	Susceptible	−	NS^c^
	C9726	Wild animal	M-Act	Lantibiotic-like	Susceptible	−	NS^c^
*S. epidermidis*	X3009	Food	M-Act	Lantibiotic-like	ERY-FA-[*msr*(A),*mph*(C)]	−	ST1025
*S. xylosus*	C9255	Wild animal	L-Act	Lantibiotic-like	PEN-[*bla*Z]	−	NS^c^
*S. hyicus*	C9585	Wild animal	M-Act	Lantibiotic-like	Susceptible	−	NS^c^
*S. simulans*	C9832	Wild animal	M-Act	Lantibiotic-like	FA-TET-[*tet*(K)]	−	NS^c^

*^a^Antimicrobial activity levels against the indicator bacteria tested (L-Act, low <20%; M-Act, medium, 20–70%; H-Act, high >70%).*

*^b^CLI, clindamycin; ERY, erythromycin; FA, fusidic acid; PEN, penicillin; SXT, trimethoprim sulfamethoxazole; TET, tetracycline.*

*^c^NS, non-studied.*

### Antimicrobial Resistance, Virulence, and Molecular Typing of the 60 BLIS^+^ Staphylococcal Isolates

The AMR phenotype/genotype and the virulence gene content results of the 60 BLIS^+^ isolates are shown in Supplementary Table 2 and summarized in Table 6. A great diversity of phenotypes and genotypes of antibiotic resistance was detected in the whole collection of 60 BLIS^+^ isolates. Among the CoNS, 30.7% showed susceptibility to all antibiotics tested, while 12.8% were MDR. Notably, clindamycin and fusidic acid were the most common AMR phenotypes observed in CoNS. The *sal*(A) gene (solely found among the *S. sciuri* isolates) and the *inu*(A) gene were detected among clindamycin-resistant isolates.

**TABLE 6:** Antimicrobial resistance phenotype/genotype and virulence gene content in the 60 BLIS^+^ isolates recovered in this study.

Species	Number of isolates	Number of isolates susceptible to all antibiotics tested	Antimicrobial resistance phenotype^a,b^	Antimicrobial resistance genotype^b^	Virulence gene content^b^
*S. pseudintermedius*	16	1	PEN^10^-OXA^1^-ERY^7^-CLI^6^-STR^5^-TET^6^- CHL^3^-SXT^6^-FA^1^	*bla*Z^10^, *mec*A^1^, *erm*(B)^7^, *aad*E^4^, *tet*(M)^6^, *cat*_pC221_^3^, *dfr*G^6^, *Inu*(A)^1^, *ant*(6)-Ia^1^	*lukS/F-I*^16^, *siet*^16^, *se-int*^12^, *sec1*^1^, *expB*^1^
*S. aureus*	5	3	PEN^2^-ERY^1^-CLI^I^ ^1^	*blaZ*^2^,*msr*(A)^1^, *erm*(C)^1^, *erm*(T)^1^, *Inu*(A)^1^	*lukMF′*^1^, *lukED*^1^, *etD2*^1^
*S. sciuri*	13	0	ERY^2^-CLI^I^ ^13^-FA^6^- TOB^1^-CIP^2^-SXT^1^	*erm*(B)^3^, *msr*(A)^1^, *sal*(A)^13^, *Inu*(A)^4^, *ant*(4’)-Ia^1^, *dfr*A^1^	−
*S. chromogenes*	10	8	CIP^1^-TET^2^	*tet*(M)^2^, *tet*(K)^1^, *tet*(L)^1^	−
*S. warneri*	6	2	PEN^3^-ERY^2^-CIP^1^-TET^2^	*bla*Z^3^, *tet*(K)^2^, *erm*(B)^2^	−
*S. epidermidis*	4	0	PEN^2^-FOX^2^-ERY^3^-SXT^1^-TET^1^-FA^2^	*mec*A*^2^*, *msr*(A)^2^, *mph*(C)^1^	−
*S. xylosus*	3	0	PEN^1^-FA^2^	*bla*Z^1^	−
*S. hominis*	1	1	Susceptible	−	−
*S. hyicus*	1	1	Susceptible	−	−
*S. simulans*	1	0	TET^1^-FA^1^	*tet*(K)^1^	−
Total CoPS	21	1	PEN^12^-OXA^1^-ERY^8^-CLI^I^ ^17^-STR^5^-TET^6^-CHL^3^-SXT^6^-FA^1^	*bla*Z^12^, *mec*A^1^, *erm*(B)^7^, *erm*(C)^1^, *erm*(T)^1^, *msr*(A)^1^, *aadE*^4^, *tet*(M)^6^, *cat*_pC221_^3^, *dfr*G^6^, *Inu*(A)^2^, *ant*(6)-Ia^1^	*lukS/F-I*^16^, *siet*^16^, *se-int*^12^, *sec1*^1^, *expB*^1^*-lukMF′*^1^, *lukED*^1^, *etD2*^1^
Total CoNS	39	12	PEN^6^-FOX^2^-ERY^7^- CLI^I^ ^13^-TOB^1^-TET^6^- CIP^4^-SXT^2^-FA^11^	*bla*Z^3^, *mec*A*^2^*, *erm*(B)^5^, *msr*(A)^3^, *mph*(C)^1^, *sal*(A)^13^, *Inu*(A)^4^, *tet*(K)^4^, *tet*(M)^2^, *tet*(L)^1^, *ant*(4’)-Ia^1^, *dfr*A*^1^*	−

*^a^PEN, penicillin; OXA, oxacillin; FOX, cefoxitin; ERY, erythromycin; CLI, clindamycin; CLI^I^, clindamycin inducible; TOB, tobramycin; STR, streptomycin; TET, tetracycline; CIP, ciprofloxacin; CHL, chloramphenicol; FUS, fusidic acid; SXT, trimethoprim–sulfamethoxazole.*

*^b^The number in superscripts indicate the number of isolates when not all isolates of the group had the same characteristics.*

In contrast, only one CoPS isolate was susceptible to all the antimicrobials tested, whereas 33.3% of them were MDR and showed mainly resistance to the following antibiotics/resistance genes: penicillin/*bla*Z, erythromycin-clindamycin/*erm*(B), and tetracycline/*tet*(M). Virulence factors were only detected among CoPS isolates, mainly in the species *S. pseudintermedius*, where the detection of the leukocidin and the *siet* and *se-int* enteroroxin genes was frequent.

## Discussion

Antimicrobial resistance is a relevant health problem worldwide that needs the development of new strategies. The human–animal–environment interface is currently being considered under the OneHealth approach to better understand the ecology and dissemination of AMR and to control the silent pandemy, as is considered by some authors (Swift et al., 2019; Penna et al., 2021). In this context, the use of probiotics or new antimicrobial compounds, such as bacteriocins, is an interesting alternative to the use of conventional antibiotics. These peptides might become important biological weapons, especially against emerging drug-resistant bacteria, and they might be used in different areas, such as the food industry, medicine, veterinary, and agriculture (de Freire Bastos et al., 2020).

The *Staphylococcus* genus has been widely described in the literature as a bacteriocin producer. Several studies have described staphylococcins in strains from food products, such as milk (Hyicin 3682, Aureocin A70, and Aureocin A53) (Netz et al., 2001; Netz et al., 2002; Carlin Fagundes et al., 2017), fermented food (Micrococcin P1 and Nukacin ISK-1) (Cundliffe and Thompson, 1981; Sashihara et al., 2000), fish (Nukacin KQU-131) (Wilaipun et al., 2008), livestock (BacCH91 and Gallidermin) (Kellner et al., 1988; Wladyka et al., 2013), and also in strains from pets (BacSp222) (Wladyka et al., 2015). However, very few studies have reported bacteriocins produced by staphylococci from wild animals, except for some studies on animals from the marine environment (Prichula et al., 2021). Regarding the available information about the environment, the soil is the most widely studied natural source of antimicrobial peptides, and *Bacillus* is the most representative genus of soil bacteriocin producers (Zimina et al., 2020).

In humans, bacteriocin production has been detected in clinical and commensal *Staphylococcus* isolated from the skin (Capidermicin, NisinJ, and Staphylococcin C55) (Dajani and Wannamaker, 1969; Lynch et al., 2019; O’Sullivan et al., 2020) and nose (Nukacin IVK45 and Lugdunin) (Janek et al., 2016; Zipperer et al., 2016).

In this study, production of BLIS was found in 6.7% of isolates of a large collection of staphylococci (both CoPS and CoNS) of many different origins (wild animal, food, pet, environment, and human) that were tested against a wide series of indicator bacteria, including MDR bacteria, and also bacteria with relevant mechanisms of AMR or with zoonotic interest. According to our results, the species with the highest percentages of BLIS^+^ isolates were *S. chromogenes* (38.5%), *S. pseudintermedius* (26.7%), and *S. warneri* (23.1%), which highlights the capacity of CoNS, usually considered as common colonizers and non-pathogenic bacteria, to compete against other bacteria, including pathogens (Janek et al., 2016; O’Sullivan et al., 2019). The percentages of BLIS^+^ isolates in other staphylococcal species were lower: *S. hyicus* (16.7%), *S. hominis* (12.5%), *S. epidermidis* (10.5%), *S. xylosus* (6.25%), *S. sciuri* (6.1%), and *S. aureus* (3.4%). *S. epidermidis* has been described as one of the most frequent bacteriocin-producing CoNS species, mainly among human isolates (Bastos et al., 2009; Janek et al., 2016). Although all human *S. epidermidis* isolates tested in our study were BLIS-negative, *S. epidermidis* was the fifth CoNS species with higher antimicrobial activity rates in our study, although all the BLIS^+^ *S. epidermidis* isolates were recovered from food.

Some authors consider bacteriocin production to be a common characteristic of bacteria since most of them can produce these antimicrobial peptides (Cotter et al., 2005). Bacteriocin production has been reported in the literature for CoPS and CoNS, and *S. aureus* and *S. epidermidis* have been highlighted as highly prevalent producer species or at least with the better-characterized bacteriocins (Bastos et al., 2009).

Statistically significant differences were detected in this study between the origin of the isolates and the production of BLIS. Considering the total collection of *Staphylococcus* tested, BLIS production was more frequently detected among isolates of pets (30%), food (6.1%), and wild animals (6.2%) (percentages obtained with respect to the number of total isolates tested in each origin); these BLIS^+^ isolates represented 25%, 28.3%, and 41.7% of the 60 BLIS^+^ isolates, respectively. The high rate of BLIS production detected among isolates of pets might be explained by the fact that all the isolates analyzed from this originally belonged to the species *S. pseudintermedius*, one of the higher BLIS^+^ species detected in our study, as previously indicated. In humans, only one of the 27 tested isolates (3.7%) showed BLIS production, and this isolate was *S. pseudintermedius*, which interestingly was obtained from the clinical sample of a human cohabitating with pets. Higher frequencies of bacteriocin production have been previously reported in nasal staphylococci of human origin (80%) when bacteria of the nasal ecosystem were used as indicator bacteria (Janek et al., 2016). A wide diversity of variables could explain the differences observed in bacteriocin production rates, but the different origins and species of the staphylococci tested for and the different indicator bacteria employed might be involved.

Most of the known bacteriocins have a narrow spectrum of activity, often active against closely related bacteria (Cotter et al., 2013); nevertheless, in relation to staphylococcins, a wide variety of antimicrobial profiles against pathogens have been described depending on the peptide (*Streptococcus*, *Enterococcus*, *Corynebacterium*, *Bacillus*, *Clostridioides*, *Klebsiella*, and *Neisseria*, among others) (de Freire Bastos et al., 2020). This might indicate that this genus needs bacteriocins as a strategy to be maintained in complex ecosystems in different hosts (Janek et al., 2016).

All Gram-positive indicator bacteria were inhibited by at least one of the BLIS^+^ staphylococci, but this did not happen with Gram-negative indicator bacteria. It was especially relevant to the antimicrobial activity detected against *S. delphini*, *S. pseudintermedius*, *C. perfringens*, and *M. luteus*. Notably, *C. perfringens* is an important pathogen in poultry production (Nhung et al., 2017), and *S. pseudintermedius* is an emerging zoonotic pathogen in humans, especially in those with close contact with dogs/cats (Lozano et al., 2017).

Interestingly, we detected 7 BLIS^+^ isolates of the species *S. aureus*, *S. pseudintermedius*, *S. sciuri*, and *S. hominis*, which presented antimicrobial activity against > 70% of the indicator bacteria, including MRSP, MRSA, VRE, *L. monocytogenes*, *C. perfringens*, or *S. suis*, among others. These BLIS^+^ isolates are of interest for further in-depth characterization.

It is known that staphylococcal strains can carry one or more gene clusters responsible for bacteriocin production (de Freire Bastos et al., 2020). In our study, 16 of the 60 BLIS^+^ isolates showed one of the bacteriocin genes analyzed. Many *Staphylococcus* isolates are producers of lantibiotics, and a variety of these bacteriocins have been described (Neubauer et al., 1999). Significantly, putative lantibiotic-biosynthetic gene clusters have been recently found in the genome of *S. capitis*, which share homology with the biosynthetic systems of epidermin/gallidermin and the non-lantibiotic bacteriocin epidermicin (Kumar et al., 2017). According to our results, structural genes encoding putative lantibiotic-like bacteriocins were detected by PCR and sequencing in 11 isolates, both CoNS (*n* = 5) and CoPS (*n* = 6). Moreover, the gene of the recently described bacteriocin BacSp222 (Wladyka et al., 2015) was detected in three of the BLIS^+^ *S. pseudintermedius* isolates, and interestingly, all showed H-Act; nevertheless, this gene was not found in the other staphylococci species tested. In addition, it is worth highlighting the detection of the gene encoding a putative circular bacteriocin (uberolysin-like) in two of our *S. chromogenes* BLIS^+^ isolates tested. The circular bacteriocins have been commonly described in *Bacillus* and have been rarely identified in *Staphylococcus* species, excepting the Aureocyclin 4185 described in *S. aureus* (Potter et al., 2014). The detection of this putative circular bacteriocin in two *S. chromogenes* isolates would be the first report of this CoNS species, indicating the possible transfer of genetic material between staphylococcal species or the possible detection of a new bacteriocin. Notably, four of our isolates with high (X3041, X3011, and C5835) or medium (X2969) antimicrobial activity lacked all the 23 bacteriocin encoding genes tested. Further studies will be performed with these isolates to determine if they produce new bacteriocins that could be of interest.

According to the AMR phenotype and genotype and the virulence content of the BLIS^+^ isolates, it was observed in this study that 30.7% of BLIS^+^ CoNS and 19% of CoPS were susceptible to all antibiotics tested; nevertheless, 12.8% and 33.3% of BLIS^+^ CoNS and CoPS isolates, respectively, were MDR. Moreover, all the CoNS lacked the virulence genes tested, while different virulence gene profiles were observed among CoPS. Currently, the use of bacteriocins or bacteriocin-producing bacteria as an alternative to antibiotics needs to consider strains that meet all safety, efficacy, and viability criteria to be used in consortium formulations, which are being proposed as the next generation of probiotics (Eveno et al., 2021). In this context, isolates lacking acquired AMR genes or virulence genes are of special relevance.

## Conclusion

Bacteriocin production is a defense strategy or adaptive mechanism that contributes to the success of niche colonization. In this study, 6.7% of the staphylococci tested were BLIS^+^, showing a wide variety of inhibition patterns in relation to the species of producer isolates and their origins. It is worth noting the interest of CoNS isolates, due to their high antimicrobial activity profiles confirmed by the detection of bacteriocin encoding genes and the lack of relevant acquired AMR or virulence genes. These results leave a gateway for further biochemical and genetic characterization of high relevance for these BLIS^+^ isolates that can be considered as excellent candidates for a further in-depth study. To conclude, the OneHealth approach is important to better understand the interactions between colonizing bacteria in specific niches and their spread to other environments, considering the molecular ecology of AMR and other mechanisms of bacterial fitness.

## Data Availability Statement

The original contributions presented in the study are included in the article/Supplementary Material, further inquiries can be directed to the corresponding author.

## Author Contributions

CT, MZ, and CL contributed to the design and the general supervision of the study. RF-F developed most of the experimental work and the first version of the manuscript. CT made the first revision of the manuscript. PE, LR-R, and IA contributed to some experimental laboratory work related to AMR genes and molecular typing. CT and MZ contributed to project funding. All authors revised the different versions of the manuscript, read, and agreed to the submitted version of the manuscript.

## Conflict of Interest

The authors declare that the research was conducted in the absence of any commercial or financial relationships that could be construed as a potential conflict of interest.

## Publisher’s Note

All claims expressed in this article are solely those of the authors and do not necessarily represent those of their affiliated organizations, or those of the publisher, the editors and the reviewers. Any product that may be evaluated in this article, or claim that may be made by its manufacturer, is not guaranteed or endorsed by the publisher.
