# Supplementary material for: Bacteriocin-Like Inhibitory Substances in Staphylococci of Different Origins and Species With Activity Against Relevant Pathogens
Source: Front Microbiol. 2022 Apr 26;13:870510. doi: 10.3389/fmicb.2022.870510 (PMC9087342; doi:10.3389/fmicb.2022.870510)
Supplement: Supplementary file 1 [file Table_1.docx]

**SUPPLEMENTAL MATERIAL**

**Table S1.** Primer sequences and PCR conditions used in this study for the detection of bacteriocin encoding genes and bacteriocin families.

| **Bacteriocin** | **Gene** | **Primer sequence (5′→3′)** | **Annealing Tª (ºC)** | **Amplicon size (bp)** | **Reference** |
| --- | --- | --- | --- | --- | --- |
| Aureocin A70 | *aurA* | F-CCTTATAACTTCGAATGCT | 50 | 525 | (Ceotto et al., 2009) |
|  |  | R-AAATATTAACAAGAGAAA |  |  |  |
| Aureocin A53 | *aucA* | F-GAAGTTGTGAAAACTATTA | 50 | 322 | (Ceotto et al., 2009) |
|  |  | R-CATAAAACAAAGAGCCAAAG |  |  |  |
| Aureocyclin 4185 | *acIA* | F-ATGTTGTTAGAGTTAACAGG | 55 | 195 | (Potter et al., 2014) |
|  |  | R-TTAAAAAGCAATACCTGCTTTTTTTCC |  |  |  |
| BacCH91 | *bacCH91* | F-TTAGTGAAAATAAATAGTA | 42^a^ | 380 | (Wladyka et al., 2013) |
|  |  | R-CATTTGTAAGCACCTCAC |  |  |  |
| BsaA2 | *bsaA2* | F-TTAACAGCAGAAGCTATTAAAACTACCAG | 50 | 144 | (Ceotto et al., 2009) |
|  |  | R-ATGGAAAAAGTTCTTGATTTAGACG |  |  |  |
| ^b^BS (BsaA1/A2  BacCH91) |  | F-GTTCTTGATTTAGACGTRCAAG | 50 | ND^c^ | Designed in this study |
|  | Family | R-CAGAAGCTRTTAAAACTACCMGTC |  |  |  |
| BacSp222 |  | F-AAAAGGTTAGGGGGAGCCA | 55 | 818 | Designed in this study |
|  |  | R-CCGCACACTTCTCTCCACTT |  |  |  |
| Capidermicin | *orf4* | F-CCGCTCACAAAGCTTAAAC | 57 | 185 | Designed in this study |
|  |  | R-CAAAATACGGTCCAAAAGCTG |  |  |  |
| Endopeptidase ALE-1 | *ale-1* | F-AGGACCATTATGGGGAACAA | 57 | 177 | Designed in this study |
|  |  | R-AACGCGTGGTTAAGGGTATG |  |  |  |
| Epidermin | *epiA* | F-GGAGTGTTTAAAATGGAAGC | 55 | 431 | (Ceotto et al., 2009) |
|  |  | R-CCTTTTCCCAGTCTATTTTG |  |  |  |
| Epidicin 280 | *eciA* | F-CGGAGGGATATATTATGG | 50 | 195 | (Ceotto et al., 2009) |
|  |  | R-CAATCACTACTATTGACAATCAC |  |  |  |
| Epilancin 15X | *elxA* | F_ATGAATAACGAATTATT(C/T)(A/G)ATTTGGATC | 52 | 139 | (Velásquez et al., 2011) |
|  |  | R-CTTTGTAGAGGATTTACACTAACTTG |  |  |  |
| Epilancin K7 | *elkA* | F-CTCAAAAGAGTGATTTAAGTCCGC | 50 | 115 | (Ceotto et al., 2009) |
|  |  | R-CCACCAGTAATATTGCAACCGC |  |  |  |
| Epidermicin NI01 | *ecdA* | F-GGCAGCATTTATGAAGTTAATTCAG | 57 | 106 | Designed in this study |
|  |  | R-ACCGGCGTTAATCCATTTTA |  |  |  |
| Gallidermin/Staphylococcin T (StT) | *gdmA* | F-AGGAGTGTTTAAAATGGAAGCA | 50 | 154 | Designed in this study |
|  |  | R-GAAGCTACCTGTTTTGGCACA |  |  |  |
| ^b^GEST (Gallidermin, Staphylococcin T (StT), Epidermin) | Family | F-RATCTTGAYGTTAAAGTWAATGC | 52 | ND^c^ | Designed in this study |
|  |  | R-TACCTGTTTTKGCACATCCA |  |  |  |
| Hyicin/Agneticin 3682 | *hyiA* | F-TATAGGAGGTTTTTTGCTATGG | 50 | 158 | (Fagundes et al., 2017) |
|  |  | R-CAACAATAACTATTGAAGCTACC |  |  |  |
| Hyicin/Agneticin 4244 | *hycS* | F-TGGAAGTCATACATGAGAGGAGG | 50 | 150 | (Duarte et al., 2018) |
|  |  | R-ACCGAATGTACCTGTGATACCC |  |  |  |
| Lugdunin | *lugD* | F-TTCGGGAACTACTGGAATGC | 57 | 189 | Designed in this study |
|  |  | R-AAATGCAATGTCCCTCCAAC |  |  |  |
| Lysostaphin | *lss* | F-CTGAGTACATTTGCCGC | 50 | 756 | (Thumm and Gotz, 1997) |
|  |  | R-GACCGTAACCATATCCT |  |  |  |
| Nisin J | *nsj* | F-ACTTTATAACTAAGATTAGC | 52 | 182-216 | (O’ Sullivan et al., 2020) |
|  |  | R-TCGCTTTATTATTTAGTATGCACG |  |  |  |
| Nukacin IVK45 | *nuk*A | F-AAAGGGGGTATTATAATGGAAAA | 57 | 202 | Designed in this study |
|  |  | R-TCCTTGCATGATTTTATCCACA |  |  |  |
| Nukacin KQU-131 | *nkqA* | F-GGAGGTAACAAACATGGAAAATTC | 50 | 259 | (Wilaipun et al., 2008) |
|  |  | R-GCCATAATATTAGTATCATGTTAATC |  |  |  |
| Nukacin ISK-1 | *nukA* | F-AGGAGGTAACAAACATGG | 50 | 195 | (Ceotto et al., 2009) |
|  |  | R-CCCCTTTTTATGAACAACAAG |  |  |  |
| Nukacin 3299 | *nukA* | Nukacin ISK-1 primer | 50 | 176 | (Ceotto et al., 2010) |
| ^b^NUK (Nukacin KQU-131,  Nukacin 3299, Nukacin ISK1) |  | F-TGAAGGACATTGAAGTAGCAAA | 57 | 121 | Designed in this study |
|  | Family | R-TTCATATGGCAATCGTGTGAC |  |  |  |
| Pep5 | *pepA* | F-AGAGGAGGTGGTTATATATG | 50 | 427 | (Ceotto et al., 2009) |
|  |  | R-TGAGTTCCATGCCCAGTG |  |  |  |
| Staphylococcin C55 | *sacaA/ sacbA* | F-AGCGTGGTGATTCTTATG | 50 | 499 | (Ceotto et al., 2009) |
|  |  | R-TCTGATTTATTTAGTTCTGGATA |  |  |  |

^a^Specific PCR conditions: 1 cycle at 94 °C for 2 min,30 cycles at 94 °C for 30 s, 42 °C for 30 s, and 72 °C for 45 s. The rest of the PCR were carried out under standard conditions based on the annhealing temperature of each primer: 1 cycle at 94 °C for 7 min,30-35 cycles at 94 °C for 1 m, Annealing T° (°C) for 1 m, 72 °C for 1 m, and 72 °C for 10 m.

^b^BS, GEST and NUK were the bacteriocin families described in this study.

^c^ND: non defined.

**REFERENCES NOT INCLUDED IN THE MAIN MANUSCRIPT**

Ceotto, H., Nascimento Jdos, S., Brito, M. A., and Bastos Mdo, C. (2009). Bacteriocin production by *Staphylococcus aureus* involved in bovine mastitis in Brazil. Res. Microbiol. 160, 592–599. doi: 10.1016/j.resmic.2009.07.007

Ceotto, H., Holo, H., da Costa, K. F. S., Nascimento Jdos, S., Salehian, Z., Nes, I. F., et al. (2010). Nukacin 3299, a lantibiotic produced by *Staphylococcus Q23 simulans* 3299 identical to nukacin ISK-1. Vet. Microbiol. 146, 124–131. doi: 10.1016/j.vetmic.2010.04.032

Duarte, A. F. S., Ceotto-Vigoder, H., Barrias, E. M., Souto-Padrón, T. C. B. S., Nes, I. F., and Bastos, M. C. F. (2018). Hyicin 4244, the first sactibiotic described in staphylococci, exhibits an anti-staphylococcal biofilm activity. Int. J. Antimicrob. Agents 51, 349–356. doi: 10.1016/j.ijantimicag.2017.06.025

Thumm, G., and Gotz, F. (1997). Studies on prolysostaphin processing and characterization of the lysostaphin immunity factor (Lif) of *Staphylococcus simulans biovar* staphylolyticus. Mol. Microbiol. 23, 1251–1255. doi: 10.1046/j. 1365-2958.1997.2911657.x

Velásquez, J. E., Zhang, X., and van der Donk, W. A. (2011). Biosynthesis of the antimicrobial peptide epilancin 15X and its N-Terminal lactate. Chem. Biol. 18, 857–867. doi: 10.1016/j.chembiol.2011.05.007
