# Supplementary material for: Bacteriocin-Like Inhibitory Substances in Staphylococci of Different Origins and Species With Activity Against Relevant Pathogens
Source: Front Microbiol. 2022 Apr 26;13:870510. doi: 10.3389/fmicb.2022.870510 (PMC9087342; doi:10.3389/fmicb.2022.870510)
Supplement: Supplementary file 2 [file Table_2.docx]

**Table S2.** Antimicrobial resistance phenotype/genotype, virulence content, bacteriocin encoding genes and molecular typing of the 60 strains producers of bacteriocin-like inhibitory substances (BLIS).

| **Strain** | **Species** | **Origin** | **Antimicrobial resistance phenotype^a^** | **Antimicrobial resistance genotype** | **Virulence genes detected** | ***Spa-*MLST-CC/ *agr*** | **Bacteriocin genes**  **detected** | **Reference** |
| --- | --- | --- | --- | --- | --- | --- | --- | --- |
| **C3869** | *S. pseudintermedius* | Pet | PEN-OXA-ERY-CLI-STR-TET-SXT | *bla*Z, *mec*A, *erm*(B),*tet*(M), *dfr*G | *luk*S/F-I*, siet, se-int* | ST258/II/ (SCC*mec*IV) | - | (Ruiz-Ripa et al. 2021) |
| **C5344** | *S. pseudintermedius* | Pet | PEN-ERY-CLI-STR-TET-CHL-SXT | *bla*Z, *erm*(B), *tet*(M), *cat*_pC221_, *dfr*G | *luk*S/F-I*, siet, se-int* | ST2171/III | - | (Ruiz-Ripa et al. 2021) |
| **C5345** | *S. pseudintermedius* | Pet | PEN-ERY-CLI-STR-TET-CHL-SXT | *bla*Z, *erm*(B),*tet*(M), *cat*_pC221_, *dfr*G | *luk*S/F-I*, siet, se-int* | ST2171/III | - | (Ruiz-Ripa et al. 2021) |
| **C3877** | *S. pseudintermedius* | Pet | PEN-STR-TET | *bla*Z,*aadE*,*tet*(M) | *luk*S/F-I*, siet, se-int, expB* | ST2172/III | - | (Ruiz-Ripa et al. 2021) |
| **C3873** | *S. pseudintermedius* | Pet | PEN | *bla*Z | *luk*S/F-I*, siet, se-int* | ST2173/III | - | (Ruiz-Ripa et al. 2021) |
| **C3874** | *S. pseudintermedius* | Pet | Susceptible | - | *luk*S/F-I*, siet, se-int* | ST2170/III | - | (Ruiz-Ripa et al. 2021) |
| **C5357** | *S. pseudintermedius* | Pet | PEN | *bla*Z | *luk*S/F-I*, siet, se-int* | ST356/III | - | (Ruiz-Ripa et al. 2021) |
| **C4502** | *S. pseudintermedius* | Pet | SXT | *dfr*G | *luk*S/F-I*, siet, se-int* | ST160/III | Lantibiotic-like | UR^c^ collection |
| **C4506** | *S. pseudintermedius* | Pet | FA | - | *luk*S/F-I*, siet, se-int,* | ST161/III | - | UR^c^ collection |
| **C4525** | *S. pseudintermedius* | Pet | PEN-ERY-CLI-STR- CHL-SXT | *bla*Z, *ant*(6)-Ia, *erm*(B), *dfr*G*, Inu*(A),*cat*_pc221_ | *luk*S/F-I*, siet, se-int* | ST162/III | - | UR^c^ collection |
| **C4486** | *S. pseudintermedius* | Pet | PEN-TET | *bla*Z, *tet*(M) | *luk*S/F-I*, siet, se-int, sec1,* | ST20/III | - | UR^c^ collection |
| **C4476** | *S. pseudintermedius* | Pet | PEN-TET-SXT | *bla*Z, *tet*(M*), dfr*G | *luk*S/F-I*, siet, se-int,* | ST160/III | - | UR^c^ collection |
| **C2912** | *S. pseudintermedius* | Pet | PEN | *bla*Z | *luk*S/F-I*, siet* | ST29/III | - | (Gómez-Sanz et al., 2013) |
| **C8189** | *S. pseudintermedius* | Human | ERY-CLI | *erm*(B) | *luk*S/F-I*, siet* | ST241/III | Bacsp222 | (Lozano et al., 2017) |
| **C8478** | *S. pseudintermedius* | Pet | ERY-CLI | *erm*(B) | *luk*S/F-I*, siet* | ST241/III | Bacsp222 | (Lozano et al., 2017) |
| **C8479** | *S. pseudintermedius* | Pet | ERY-CLI | *erm*(B) | *luk*S/F-I*, siet* | ST241/III | Bacsp222 | (Lozano et al., 2017) |
| **C5802** | *S. aureus* | Environmental | PEN | *bla*Z | *luk*MF*´, luk*ED*, etD2* | t843-ST130-CC130/III | Lantibiotic-like | (Gómez et al., 2017) |
| **C6770** | *S. aureus* | Wild animal | Susceptible | - | - | t1125-CC5/II | Lantibiotic-like | (Ruiz-Ripa et al., 2019) |
| **C8609** | *S. aureus* | Wild animal | Susceptible | - | - | t11225-CC425/II | Lantibiotic-like | (Ruiz-Ripa et al., 2019) |
| **X3410** | *S. aureus* | Food | Susceptible | - | - | t10234/I | Lantibiotic-like | This study |
| **X3417** | *S. aureus* | Food | PEN-ERY-CLI^I^ | *bla*Z*, mrs*(A)*, erm*(C)*, ermT, Inu*(A) | - | t1451-CC398/I (IEC+C) | - | This study |
| **C9175** | *S. sciuri* | Wild animal | CLI^I^ | *Inu*(A),*sal*(A) | - | - | - | (Ruiz-Ripa et al., 2020) |
| **C9179** | *S. sciuri* | Wild animal | CLI^I^ | *sal*(A) | - | - | - | (Ruiz-Ripa et al., 2020) |
| **C9185** | *S. sciuri* | Wild animal | CLI^I^-FA | *sal*(A) | - | - | - | (Ruiz-Ripa et al., 2020) |
| **C9188** | *S. sciuri* | Wild animal | CLI^I^ | *Inu*(A),*sal*(A) | - | - | - | (Ruiz-Ripa et al., 2020) |
| **C9191** | *S. sciuri* | Wild animal | CLI^I^ | *sal*(A) | - | - | - | (Ruiz-Ripa et al., 2020) |
| **C9193** | *S. sciuri* | Wild animal | CLI-FA | *Inu*(A),*sal*(A) | - | - | - | (Ruiz-Ripa et al., 2020) |
| **C9203** | *S. sciuri* | Wild animal | CLI^I^ | *sal*(A) | - | - | - | (Ruiz-Ripa et al., 2020) |
| **C9213** | *S. sciuri* | Wild animal | CLI^I^-FA | *sal*(A) | - | - | - | (Ruiz-Ripa et al., 2020) |
| **C9231** | *S. sciuri* | Wild animal | CLI^I^-FA | *sal*(A) | - | - | - | (Ruiz-Ripa et al., 2020) |
| **C9258** | *S. sciuri* | Wild animal | ERY CLI -TOB-CIP-FA | *erm*(B), *msr*(A), *sal*(A), *ant*(4’)-Ia | - | - | - | (Ruiz-Ripa et al., 2020) |
| **C9529** | *S. sciuri* | Wild animal | CLI | *sal*(A) | - | - | - | (Ruiz-Ripa et al., 2020) |
| **X3011** | *S. sciuri* | Food | ERY-CLI-CIP-SXT | *sal*(A)*, dfr*A*, erm*(B) | - | - | - | This study |
| **X3041** | *S. sciuri* | Food | CLI-FA | *sal*(A)*, erm*(B)*, Inu*(A) | - | - | - | This study |
| **C9838** | *S. chromogenes* | Wild animal | Susceptible | - | - | - | Uberolysin-like | (Mama et al., 2019) |
| **C9853** | *S. chromogenes* | Wild animal | Susceptible | - | - | - | - | (Mama et al., 2019) |
| **C9726** | *S. chromogenes* | Wild animal | Susceptible | - | - | - | Lantibiotic-like | (Mama et al., 2019) |
| **C9727** | *S. chromogenes* | Wild animal | Susceptible | - | - | - | Lantibiotic-like | (Mama et al., 2019) |
| **C9567** | *S. chromogenes* | Wild animal | Susceptible | - | - | - | - | (Mama et al., 2019) |
| **C9581** | *S. chromogenes* | Wild animal | Susceptible | - | - | - | Uberolysin-like | (Mama et al., 2019) |
| **C9826** | *S. chromogenes* | Wild animal | Susceptible | - | - | - | - | (Mama et al., 2019) |
| **X3283** | *S. chromogenes* | Food | Susceptible | - | - | - | - | This study |
| **X3300** | *S. chromogenes* | Food | TET | *tet*(M)*, tet*(K) | - | - | - | This study |
| **X3390** | *S. chromogenes* | Food | TET-CIP | *tet*(L)*, tet*(M) | - | - | - | This study |
| **X3007** | *S. warneri* | Food | PEN-ERY | *blaZ*, *erm*(B) | - | - | - | This study |
| **X3015** | *S. warneri* | Food | PEN-ERY | *blaZ*, *erm*(B) | - | - | - | This study |
| **X3023** | *S. warneri* | Food | Susceptible | - | - | - | - | This study |
| **X2969** | *S. warneri* | Food | Susceptible | - | - | - | - | This study |
| **X3027** | *S. warneri* | Food | TET | *tet*(K) | - | - | - | This study |
| **X3044** | *S. warneri* | Food | PEN-TET-CIP | *bla*Z, *tet*(K) | - | - | - | This study |
| **X3009** | *S. epidermidis* | Food | ERY-FA | *msr*(A)*, mph*(C) | - | ST1025^b^ | Lantibiotic-like | This study |
| **X3026** | *S. epidermidis* | Food | ERY | *erm*(B) | - | - | - | This study |
| **X3047** | *S. epidermidis* | Food | PEN-FOX-ERY-SXT | *msr*(A)*, mec*A | - | - | - | This study |
| **X3353** | *S. epidermidis* | Food | PEN-FOX-TET-FA | *mec*A | - | - | - | This study |
| **C9255** | *S. xylosus* | Wild animal | PEN | *bla*Z | - | - | Lantibiotic-like | (Ruiz-Ripa et al., 2020) |
| **C9576** | *S. xylosus* | Wild animal | FA | - | - | - | - | (Mama et al., 2019) |
| **C9793** | *S. xylosus* | Wild animal | FA | - | - | - | - | (Mama et al., 2019) |
| **C5835** | *S. hominis* | Environmental | Susceptible | - | - | - | - | (Gómez et al., 2017) |
| **C958*5*** | *S. hyicus* | Wild animal | Susceptible | - | - | - | Lantibiotic-like | (Mama et al., 2019) |
| **C9832** | *S. simulans* | Wild animal | TET-FA | *tet*(K) | - | - | Lantibiotic-like | (Mama et al., 2019) |

^a^PEN, penicillin; OXA; oxacillin; FOX, cefoxitin; ERY, erythromycin; CLI, clindamycin; CLI^I^, clindamycin inducible; TOB, tobramycin; STR, streptomycin; TET, tetracycline; CIP, ciprofloxacin; CHL, chloramphenicol; SXT, trimethoprim–sulfamethoxazole; FA, fusidic acid.

^b^Strain typed as ST1025. The MLST characterization was carried out exclusively in this strain due to their interesting antimicrobial activity spectra evaluated by the *spot-on-lawn* method and the lack of bacteriocin coding genes.

^c^UR: University of La Rioja.
