# Supplementary material for: Bacteriocin-Like Inhibitory Substances in Staphylococci of Different Origins and Species With Activity Against Relevant Pathogens
Source: Front Microbiol. 2022 Apr 26;13:870510. doi: 10.3389/fmicb.2022.870510 (PMC9087342; doi:10.3389/fmicb.2022.870510)
Supplement: Supplementary file 3 [file Image_1.pdf]

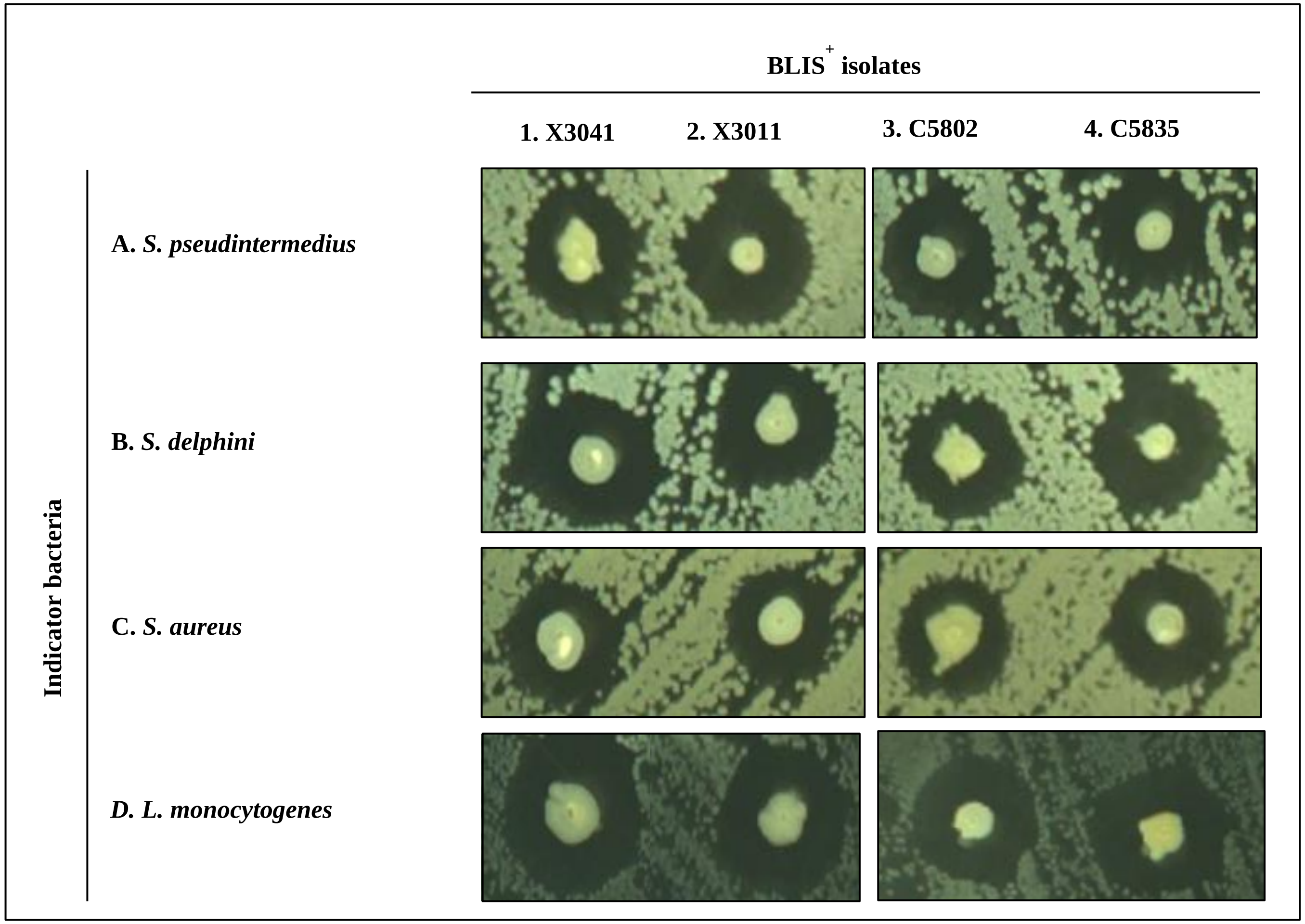

Figure S1. Antimicrobial activity of four BLIS^{+} staphylococcal isolates against different indicator bacteria by the *spot-on-lawn* method.
